# The putative oncogene, *CRNDE,* is a negative prognostic factor in ovarian cancer patients

**DOI:** 10.18632/oncotarget.6016

**Published:** 2015-11-04

**Authors:** Lukasz Michal Szafron, Anna Balcerak, Ewa Anna Grzybowska, Barbara Pienkowska-Grela, Agnieszka Podgorska, Renata Zub, Magdalena Olbryt, Jolanta Pamula-Pilat, Katarzyna M. Lisowska, Ewa Grzybowska, Tymon Rubel, Agnieszka Dansonka-Mieszkowska, Bozena Konopka, Magdalena Kulesza, Martyna Lukasik, Jolanta Kupryjanczyk

**Affiliations:** ^1^ Department of Pathology and Laboratory Diagnostics, Maria Sklodowska-Curie Memorial Cancer Center and Institute of Oncology, Warsaw, Poland; ^2^ Department of Molecular and Translational Oncology, Maria Sklodowska-Curie Memorial Cancer Center and Institute of Oncology, Warsaw, Poland; ^3^ Center for Translational Research and Molecular Biology of Cancer, Maria Sklodowska-Curie Memorial Cancer Center and Institute of Oncology, Gliwice Branch, Poland; ^4^ Institute of Radioelectronics and Multimedia Technology, Warsaw University of Technology, Warsaw, Poland

**Keywords:** ovarian cancer, prognostic factor, CRNDE, gene expression, TP53

## Abstract

The *CRNDE* gene seems to play an oncogenic role in cancers, though its exact function remains unknown. Here, we tried to assess its usefulness as a molecular prognostic marker in ovarian cancer. Based on results of our microarray studies, *CRNDE* transcripts were further analyzed by Real-Time qPCR-based profiling of their expression. The qPCR study was conducted with the use of personally designed TaqMan assays on 135 frozen tissue sections of ovarian carcinomas from patients treated with platinum compounds and either cyclophosphamide (PC, *N* = 32) or taxanes (TP, *N* = 103). Elevated levels of two different *CRNDE* transcripts were a negative prognostic factor; they increased the risk of death and recurrence in the group of patients treated with TP, but not PC (DNA-damaging agents only). Higher associations were found for overexpression of the short *CRNDE* splice variant (FJ466686): HR 6.072, 95% CI 1.814–20.32, *p* = 0.003 (the risk of death); HR 15.53, 95% CI 3.812–63.28, *p* < 0.001 (the risk of recurrence). Additionally, accumulation of the TP53 protein correlated with decreased expression of both *CRNDE* transcripts in tumor cells. Our results depict *CRNDE* as a potential marker of poor prognosis in women with ovarian carcinomas, and suggest that its significance depends on the therapeutic regimen used.

## INTRODUCTION

The *CRNDE* (Colorectal Neoplasia Differentially Expressed, formerly known as *LOC388279* or *LOC643911*) gene is located to the long arm of chromosome 16 (16q12.2) in human. Until recently, *CRNDE* had been treated as a long non-coding RNA (lncRNA)-coding gene [1], though we have lately identified its protein product, CRNDEP [2]. Our interest in this gene started from its identification as one of several (and the most strongest) potential prognostic factors in ovarian cancer patients [3]. Members of our group have established complete sequences of two new *CRNDE* transcripts (FJ466685 and FJ466686, published in GenBank in 2008) by using the FirstChoice RLM-RACE kit (Ambion, Carlsbad, CA, USA) (see supplementary data and Figures S1–S4 in the Supplement). One of the first published studies on *CRNDE* was an *in silico* experiment that used the boolean-based systems biology approach to predict novel genes associated with colorectal cancer [4]. The researchers identified *CRNDE* as the gene highly upregulated (F*C* = 16) in colon cancer compared with the normal colonic mucosa. Additionally, some *in vitro* studies showed that *CRNDE* is overexpressed in colorectal carcinomas and other solid tumors and leukemias [5]. The authors also presented preliminary results showing that the level of *CRNDE* lncRNAs in patients’ blood plasma increased specifically at early stages of colon cancer development. Accordingly, it was recently discovered that *CRNDE* may promote growth and invasion of glioma cells both *in vitro* and *in vivo* [6, 7]. We have shown that the CRNDEP peptide localizes predominantly to the nucleus and its expression is elevated in highly proliferating tissues [2]. All these findings make the *CRNDE* gene a promising candidate for a new biomarker of carcinogenesis.

Here, we aimed to elucidate how the expression of the two *CRNDE* transcripts identified by our team affects ovarian cancer prognosis in patients treated with two different chemotherapy regimens. Another aspect of this study was to analyze *CRNDE* expression with respect to TP53 accumulation status in the nuclei of tumor cells. TP53 accumulation is one of the most frequently observed aberrations in ovarian carcinomas; it occurs as a result of *TP53* gene alterations that affect TP53 transactivation capabilities (mainly missense mutations) [8]. This phenomenon is predominantly due to the lack of TP53 degradation in proteasomes. Mutant TP53 exerts a dominant-negative effect on the wild-type TP53, leading to a complete loss of the TP53 function [9]. Several studies on cell lines have shown that the levels of different proteins depend on the function or level of the TP53 protein. In addition, the results obtained in recent years by our group suggest that the TP53 accumulation status may influence the clinical importance of other molecular factors [10–12]. Remarkably, this is the first study investigating a clinical importance of the *CRNDE* gene in cancer patients.

## RESULTS

### Evaluation of the clinical importance of the *CRNDE* gene by gene expression microarrays, and further confirmation with real-time qPCR

The *CRNDE* gene was chosen for evaluation of its prognostic value based on the results of our analysis of gene expression microarray data that are publicly available in the Gene Expression Omnibus (GEO) database (accession number GSE63885). This microarray study revealed a strong negative impact of *CRNDE* expression on overall survival of ovarian cancer patients treated with the taxane/platinum (TP) regimen (see Table S1 in the Supplement). With FC values exceeding 5, *CRNDE* was the most prominent prognostic marker in that group and in its subgroup with TP53 accumulation. Moreover, this association was observed for both CRNDE-specific probe sets available in this microarray, 238021_s_at and 238022_at. A subsequent multivariate Cox analysis performed in the microarray group (*N* = 37), as well as in an adequate independent validation group (*N* = 66), confirmed the microarray outcome (see Table S2). The same statistical inference was conducted in the merged TP-treated group of 103 tumors, and the results turned out to be more significant than in either of the subgroups (see Table 1). This analysis also showed a negative impact of *CRNDE* overexpression on disease-free survival of patients. Remarkably, the outcome obtained for the joined platinum/cyclophosphamide (PC) and TP-treated groups was, in general, less significant than for the TP-treated group only. This accorded with the lack of significant results in the univariate Cox analysis performed for the PC-treated group (data not shown).

**Table 1:** Evaluation of a prognostic value of the *CRNDE* gene expression in the TP-treated group and the joined PC- and TP-treated groups of ovarian cancer patients

TP regimen						
			The TP53 (+) subgroup		The TP53 (−) subgroup	
Variable name	OS (87/103)*	DFS (64/75)*	OS (52/65)*	DFS (41/49)*	OS (35/38)*	DFS (23/26)*
	HR [95% CI] p	HR [95% CI] p	HR [95% CI] p	HR [95% CI] p	HR [95% CI] p	HR [95% CI] p
***CRNDE* (short variant) high vs low expr.**	6.072 [1.814–20.32] 0.003	15.53 [3.812–63.28] < 0.001	10.20 [2.007–51.80] 0.005	9.891 [1.426–68.60] 0.020	5.635 [0.839–37.86] 0.075	18.65 [1.661–209.3] 0.018
**Age ≥53 vs <53 years**	-	1.648 [0.938–2.895] 0.082	-	2.080 [0.955–4.530] 0.065	-	-
**Type (serous vs non-serous)**	-	0.446 [0.216–0.924] 0.030	-	0.393 [0.168–0.920] 0.031	2.371 [0.869–6.467] 0.092	-
**Rt >2cm vs 0cm**	3.172 [1.579–6.371] 0.001	-	3.052 [1.298–7.176] 0.011	-	-	9.874 [1.774–54.97] 0.009
**Rt ≤2cm vs 0cm**	2.494 [1.374–4.525] 0.003	1.875 [1.079–3.258] 0.026	2.774 [1.280–6.010] 0.010	-	-	2.816 [0.888–8.924] 0.079
**FIGO IIIA-IIIB vs (IIB+IIC)**	-	-	-	8.800 [0.834–92.88] 0.070	-	-
**FIGO IIIC vs (IIB+IIC)**	-	-	-	5.686 [0.725–44.61] 0.098	-	-
**FIGO IV vs (IIB+IIC)**	-	5.474 [1.614–18.56] 0.006	-	100.4 [6.951–1450] 0.001	-	-
**Grade 3 vs (1+2)**	-	2.168 [1.177–3.994] 0.013	-	2.711 [1.288–5.706] 0.009	-	-
**Grade 4 vs (1+2)**	-	-	-	-	9.300 [2.723–31.76] < 0.001	-
***CRNDE* (long variant) high vs low expr.**	6.908 [1.749–27.28] 0.006	8.760 [2.034–37.73] 0.004	7.519 [1.027–55.03] 0.047	NS	6.630 [0.899–48.93] 0.064	NS
**Rt >2cm vs 0cm**	3.170 [1.578–6.367] 0.001	-	3.110 [1.326–7.293] 0.009		-	
**Rt ≤2cm vs 0cm**	2.642 [1.465–4.765] 0.001	1.822 [1.061–3.129] 0.030	2.984 [1.381–6.445] 0.005		-	
**FIGO IV vs (IIB+IIC)**	-	4.477 [1.438–13.94] 0.010	-		-	
**Grade 3 vs (1+2)**	-	1.648 [0.976–2.782] 0.062	-		-	
**Grade 4 vs (1+2)**	-	-	-		4.911 [1.824–13.227] 0.002	
**PC+TP regimens**						
			**The TP53 (+) subgroup**		**The TP53 (−) subgroup**	
**Variable name**	OS (118/135)*	DFS (84/97)*	OS (72/86)*	DFS (54/63)*	OS (46/49)*	DFS (30/34)*
	**HR [95% CI] p**	**HR [95% CI] p**	**HR [95% CI] p**	**HR [95% CI] p**	**HR [95% CI] p**	**HR [95% CI] p**
***CRNDE* (short variant) high vs low expr.**	5.437 [1.699–17.40] 0.004	11.47 [2.869–45.84] 0.001	9.454 [2.099–42.58] 0.003	NS	NS	22.44 [2.160–233.3] 0.009
**Age ≥53 vs <53 years**	-	-	-			2.668 [1.140–6.240] 0.024
**Rt >2cm vs 0cm**	2.908 [1.674–5.051] < 0.001	-	3.325 [1.696–6.517] < 0.001			-
**Rt ≤2cm vs 0cm**	2.503 [1.502–4.169] < 0.001	1.495 [0.947–2.360] 0.084	2.432 [1.279–4.623] 0.007			-
**FIGO IV vs (IIB+IIC)**	-	4.069 [1.576–10.51] 0.004	-			9.950 [1.783–55.53] 0.009
**Grade 3 vs (1+2)**	1.688 [0.911–3.129] 0.096	-	-			-
**Grade 4 vs (1+2)**	1.853 [0.947–3.624] 0.072	-	-			4.224 [1.201–14.862] 0.025
***CRNDE* (long variant) high vs low expr.**	2.414 [0.971–6.005] 0.058	2.755 [0.863–8.800] 0.087	6.306 [1.485–26.77] 0.013	NS	NS	NS
**Age ≥53 vs <53 years**	-	1.482 [0.937–2.344] 0.093	-			
**Rt >2cm vs 0cm**	2.900 [1.675–5.021] < 0.001	-	3.568 [1.819–6.998] < 0.001			
**Rt ≤ 2cm vs 0cm**	2.413 [1.470–3.958] < 0.001	1.565 [0.992–2.469] 0.054	2.631 [1.388–4.986] 0.003			
**FIGO IV vs (IIB+IIC)**	1.950 [1.023–3.717] 0.042	5.946 [2.259–15.65] < 0.001	-			

* Values before and after a slash (/) stand for the number of completed observations vs all observations, respectively. Only the expression results with *p*-values < 0.1 are shown and those with *p*-values < 0.05 are underlined. HR and CI stand for the hazard ratio and 95% confidence interval, respectively. NS – a non-significant result. The multivariate Cox proportional hazards model was utilized in this analysis.

The clinical importance of overexpressing the short *CRNDE* splice variant (FJ466686) was particularly visible with regard to the risk of death (HR 6.072, 95% CI 1.814–20.32, *p* = 0.003) and recurrence (HR 15.53, 95% CI 3.812–63.28, *p* < 0.001) in all TP-treated patients analyzed (see Table 1).

**Figure 1:**
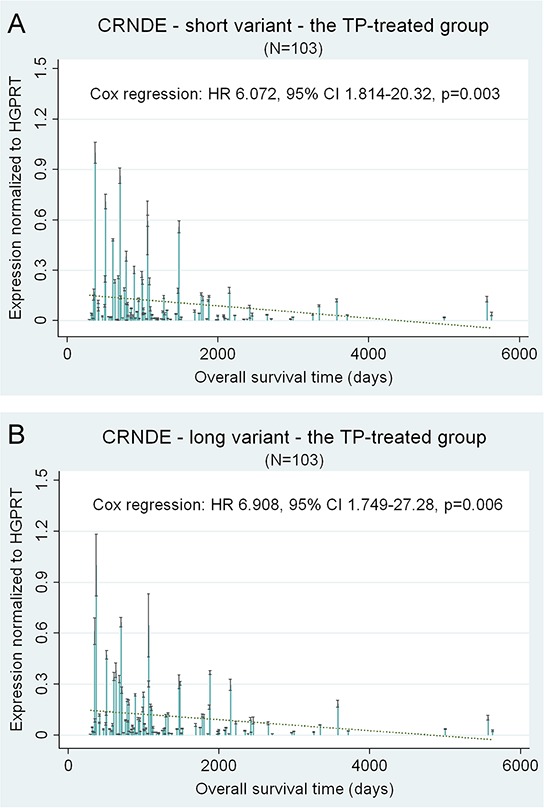
*CRNDE* – selected prognostic results of the multivariate statistical analysis of gene expression *CRNDE* expression is shown as a continuous variable. Black lines on bars represent standard deviations of Real-Time qPCR measurements for each tumor. The linear regression lines (green and dotted) are shown to visualize trends of expression.

The results obtained for the longer splice variant of *CRNDE* (FJ466685) remained in accordance with those obtained for the shorter one, showing the negative impact of *CRNDE* overexpression on overall survival (HR 6.908, CI 1.749–27.28, *p* = 0.006) and disease-free survival (HR 8.760, CI 2.034–37.73, *p* = 0.004) in the TP-treated group. However, the associations with clinical endpoints were less noticeable for the longer transcript than for the shorter one. Exemplary results are depicted in Figure 1.

**Figure 2:**
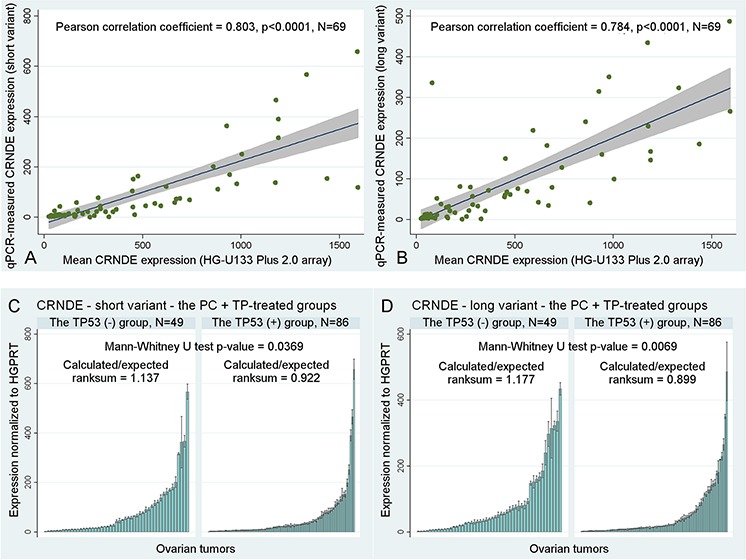
Other statistical results **A–B.** A correlation between the mean *CRNDE* expression measured with gene expression microarrays for two CRNDE-specific probe sets, 238021_s_at and 238022_at, and the results obtained in Real-Time qPCR studies with the use of our personally designed TaqMan assays for two different splice variants of *CRNDE*. Fitted regression lines are marked blue, whereas shaded regions represent the 95% confidence interval. **C–D.** The association between the accumulation of TP53 and the decreased expression of *CRNDE*. If the expression decreases, the calculated/expected ratio of the Mann-Whitney U test is lower than 1 and *vice versa*. Black lines on bars represent standard deviations of Real-Time qPCR measurements for each tumor.

Some clinical associations of *CRNDE* appeared to be dependent on the TP53 accumulation status, which was particularly visible in the validation group (see Table S2), nevertheless, the difference could also be due to unequal sizes of subgroups. We discovered a statistically significant correlation between accumulation of the TP53 protein and the decreased expression of both *CRNDE* transcripts in ovarian carcinomas (Mann-Whitney U test *p* = 0.0369, and *p* = 0.0069, for the short and long transcript, respectively, see Figure 2C–2D).

We did not find any associations between *CRNDE* expression and the following clinicopathological parameters: patient's age, histological grade and type of a tumor, and the FIGO stage.

Lastly, we have also compared the results of *CRNDE* expression profiling obtained with gene expression microarrays and Real-Time qPCR, and calculated the Pearson correlation coefficients between them. The high correlation coefficients (ranging from 0.784 to 0.803) in combination with low *p*-values < 0.0001 suggest that both aforementioned methods produced highly similar outcomes, which indirectly corroborates reliability of the results presented above (see Figure 2A–2B).

### Identification of other possible splice variants of *CRNDE*

**Figure 3:**
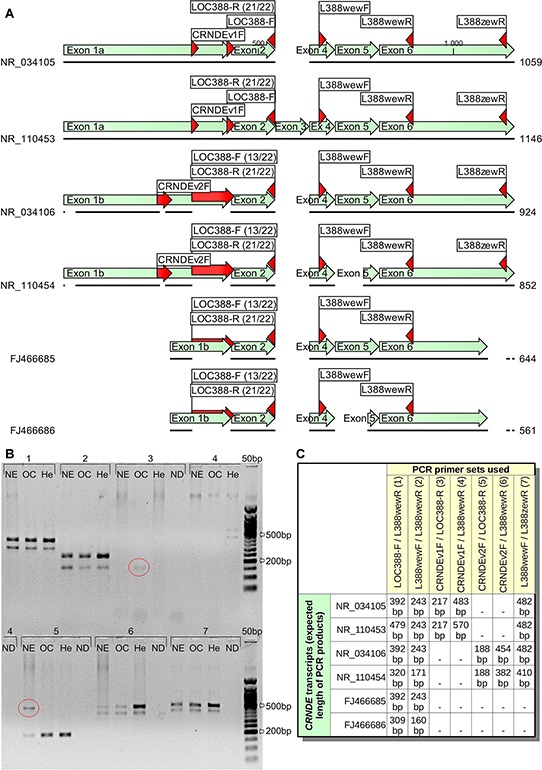
Different *CRNDE* transcripts seem to occur in a tissue-dependent manner **A.** A graphical alignment of two *CRNDE* transcripts investigated in this study (FJ466685, FJ466686) to four reference RNA sequences (NR_034105, NR_034106, NR_110453, NR_110454) available in GenBank. Regions, where the PCR primers hybridize, are marked with red arrows. **B.** Three various tissues were tested in this PCR experiment: normal endometrium (NE), ovarian cancer (OC) and HeLa cells (He). The bands characteristic for certain tissues only and also inconsistent with *in silico* predictions were marked with ellipses (see Figure C for details on the primer sets used and the expected length of PCR products). 50 bp DNA Ladder (New England Biolabs, Ipswich, MA, USA) was utilized as a size standard. The annealing temperature was set to 55°C, while the elongation time lasted for 2 minutes to allow amplification of products up to 2 kb. ND stands for a no-DNA sample. NDs for the primer sets no. 1 and 2 were not shown due to the lack of empty lanes in the gel (they were both negative).

Four reference *CRNDE* transcripts, NR_034105, NR_034106, NR_110453, and NR_110454 are spliced differently than the transcripts identified in our study (see Figure 3A). This may suggest that *CRNDE* pre-RNA is processed in many ways, depending on, e.g., a tissue type. As a result, a variety of distinct *CRNDE* RNAs could exist in different tissues. To verify this hypothesis, we carried out an additional PCR experiment on three cDNA samples derived from normal endometrium (NE), ovarian cancer (OC) and HeLa cells (He). In this study, 7 sets of primers were used (see Figure 3B–3C and Table S3). The CRNDEv1F primer was specific to the NR_034105 and NR_110453 sequences, whereas CRNDEv2F should hybridize to the NR_034106 and NR_110454 transcripts only. The expected length of PCR products, depending on the primer set used and the *CRNDE* splice variant, is shown in Figure 3C. Although the overall band pattern was in step with our earlier predictions *in silico*, some unexpected bands (marked with ellipses) emerged in a tissue-dependent manner. On the contrary, some other bands, specific to the 5′ end of the NR_034105 and NR_110453 transcripts did not appear in the gel, suggesting the lack of these splice variants in the analyzed tissues. Remarkably, the FJ466686 (shorter) transcript described herein, seemed to be present in all three samples evaluated (see double bands in Figure 3B). Accordingly, we have recently proven the prevalence of this CRNDEP-coding splice variant by measuring and comparing its expression between 21 normal human tissue sets [2].

## DISCUSSION

Herein, by performing microarray and Real-Time qPCR studies, we have shown that the elevated level of *CRNDE* transcripts significantly increases the risk of death and recurrence in ovarian cancer patients. This association was observed predominantly in patients treated with taxanes (and not in the group treated with DNA-damaging agents only), which may be a hint that this gene is somehow involved in the metabolism of microtubules during cell division. In qPCR experiments, we investigated the expression of two different splice variants of the *CRNDE* gene. The longer variant (FJ466685) contains 83 additional nucleotides in exon 5. Their absence in the shorter variant (FJ466686) leads to a formation of a new ORF, which encodes the 84 amino acid-peptide CRNDEP, recently described by our group as overexpressed in highly proliferating tissues [2]. This observation is concordant with the hypothesis by Ellis et al. [13], that *CRNDE* may be a target of the MYC regulatory pathway, being associated with the cell cycle progression and malignant transformation [14]. Interestingly, our clinical results obtained for the shorter splice variant (FJ466686, CRNDEP-coding) were statistically more significant than those for the longer one, which may imply that the former variant exhibits a higher biological activity.

The clinical importance of *CRNDE* expression was best visible in all TP-treated patients than in either the microarray or validation subgroup. This was likely due to the relatively small size of both subgroups. Nevertheless, we obtained consistent and statistically significant results for all three sample sets (see Tables 1 and S2), and the overall clinical effect seemed to be additive. These results were obtained in the multivariate Cox analysis, which strongly supports the hypothesis that *CRNDE* overexpression may be an independent, negative prognostic factor in ovarian carcinogenesis. Furthermore, the clinical significance of this novel marker seems to hinge on the therapeutic regimen used.

In accordance with our results, other researchers reported *CRNDE* overexpression in more than 90% of colorectal adenocarcinomas [5]. The authors also suggested that *CRNDE* may be a promising tissue and plasma biomarker in this cancer. Noteworthy, both splice variants identified by our team were shown by Graham et al. [5] to be highly elevated in adenomas and adenocarcinomas, but not in the normal colonic tissue. The same team reported in another article [13] that *CRNDE* may be involved in development of cancers in the variety of tissues and organs, like: colon, liver, kidney, pancreas, prostate, ovary, blood and brain. Based on some preliminary knock-in and knock-down experiments, the authors concluded that *CRNDE* transcripts can promote carcinogenesis by stimulating cell growth and suppressing apoptosis. The rise of *CRNDE* levels during cancer development may be due to the alterations in upstream signaling pathways, including the MAP kinase pathway. Recently, the same authors reported the existence of a few nuclear, intronic *CRNDE* transcripts, and found the association between expression of one of such transcripts (called gVC-In4) and the insulin/insulin-like growth factor (IGF) signaling. This mechanism seemed to depend on the activation of both the PI3K and MAPK pathways [15]. Accordingly, it was recently discovered that overexpression of the *CRNDE* gene may suppress apoptosis and promote growth and invasion of gliomas both *in vitro* and *in vivo* either in a manner depending on the mammalian Target of Rapamycin (mTOR) pathway [6] or by inhibiting the expression of the tumor-suppressing miR-186 [7]. In line with this, Chen et al. [16] reported abundant expression of CRNDE in recurrent gliomas. All these results support our findings described herein.

We have previously shown on large groups of patients that the TP53 accumulation status determined the clinical significance of altered expression of some genes, like *BAX*, *BCL2* or *ERBB2*, and polymorphisms in others (*AR, FSHR*) in ovarian cancer patients [10, 11, 17, 18]. This idea is based on the assumption, and some facts from studies on cell lines, that mutant TP53, in contrast to the wt protein, creates a “permissive” environment for action of oncogenes [19, 20]. However, this was not apparent with regard to the *CRNDE* gene. Concomitantly, the Real-Time qPCR results presented here imply an association between TP53 accumulation and lower *CRNDE* expression. In accordance with this observation, Ellis et al. [13] proposed that the *CRNDE* and *IRX5* genes may share the same bidirectional promoter, whereas the expression of *IRX5* was shown to negatively correlate with mutant TP53 protein levels in LNCaP prostate cancer cells [21].

Finally, we want to present some considerations related to the methodical part of the study. In order to achieve the highest possible specificity and sensitivity of the qPCR study, we designed and tested our TaqMan assays as recommended by PE Biosystems [22]. One of the most important factors that could potentially affect the expression analysis was the presence of single-nucleotide polymorphisms (SNPs) in the regions, where the *CRNDE*-specific primers and the TaqMan probe bind. According to the NCBI dbSNP database, there are no SNPs in the sequences recognized by two primers, LOCrtF and LOCrt35R (see Table S3). By contrast, the LOCrt4wR primer binds to a region, where one SNP, rs575423185, has been identified in the 1000 Genomes Project [23]. The Minor Allele Frequency (MAF) of this SNP in the default global population equals 0.04%. The *CRNDE*-specific TaqMan probe, that we designed, lies within a region with three known SNPs, rs572170005, rs192990372, and rs141935748. Their frequencies in the default global population equal 0.02%, 0.06% and 0.42%, respectively. This means that the probability of being a carrier of the most frequent of these SNPs (rs141935748) in at least one allele is approximately 0.0042^2 + 2*0.0042*(1 – 0.0042) ≈ 0.0084. In other words, one of about 119 (1/0.0084) individuals is a carrier of this SNP in the default global population. Considering that the analyzed group in our Real-Time qPCR study consisted of 135 ovarian cancer patients, it is highly probable that only one tumor harbored the rs141935748 polymorphism, which should not affect the *CRNDE* expression profiling in the entire group. The 3 other SNPs mentioned above are much less frequently distributed in the default global population, so they are likely absent in our group of ovarian cancer patients.

**Figure 4:**
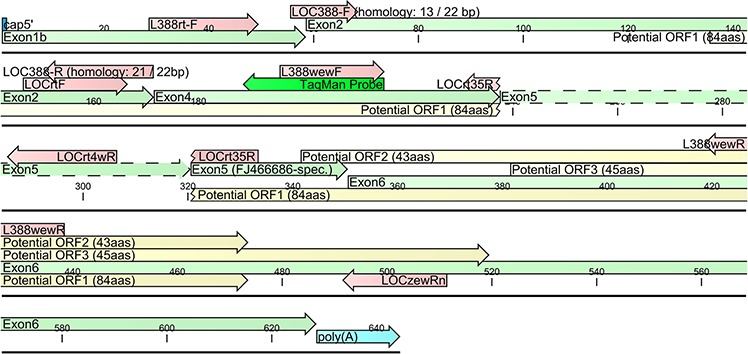
A diagram of two *CRNDE* transcripts, FJ466685 and FJ466686, investigated herein An alternatively spliced region within exon 5 was depicted with dashed lines. The presence of this region leads to a disruption of 84aa ORF encoding the CRNDEP peptide. Red arrows represent primer sequences.

As to the specificity of our TaqMan assays, they were designed in such a way to allow specific discrimination between the CRNDEP-coding (FJ466686) and other CRNDEP-non-coding transcripts, e.g. FJ466685. This goal was achieved by using the LOCrt35R reverse primer, specific to the CRNDEP-coding transcript only. By contrast, the LOCrtF universal forward primer as well as the TaqMan probe did not discriminate the *CRNDE* transcripts (see Figure 4), whereas the LOCrt4wR primer was able to detect the FJ466685 splice variant as well as all reference transcripts except one, NR_110454. Thus, our qPCR results for the CRNDEP-coding transcript are fully specific, while the results for the longer *CRNDE* splice variant, FJ466685, are, in fact, the sum of expression for several transcripts. This is worth noting in light of the findings by Graham et al. [5], who showed that *CRNDE* transcripts are differentially expressed in colorectal cancer. Nevertheless, all splice variants detected by our TaqMan assays were upregulated in that neoplasm, which is consistent with the results shown herein.

As to the diversity of *CRNDE* transcripts in human cells, in July 2015, there were 12 different *CRNDE* mRNAs collected in the AceView database, the length of which ranged from 101 bp (FN117285, identified in breast carcinoma) to 2221 bp (the variant reconstructed from 25 cDNA clones originating from various normal and cancerous tissues). Here, we compared the splice variants described by us with four reference transcripts, NR_034105, NR_034106, NR_110453, and NR_110454. Although our *CRNDE* transcripts were capped and polyadenylated, which suggested their completeness, the alignment showed that they were shorter on both ends than the reference sequences. The boundaries of exons 1a and 1b described by Graham et al. [5] represent the most external transcription initiation sites, however, the most common initiation sites are located nearer the 3′ ends of these exons. Accordingly, the 5′ end of the transcripts cloned by our group was among those mapped by 5′ RACE in colorectal cancer cell lines by Graham et al. [5]. As to the 3′ end of *CRNDE* transcripts, the polyadenylation site identified by our group was also the one, which was the most commonly observed by Graham et al., though in the reference sequences the most external polyadenylation site was indicated [5]. Consistently, one of our bacterial clones, harboring the products of 3′ RACE for *CRNDE*, had the 3′ UTR longer by 5 bases than the others, (see Figure S1). Moreover, the presence of unexpected PCR products in Figure 3B suggests that some *CRNDE* transcripts in normal endometrium and ovarian cancer cells are likely spliced in a different, currently undescribed way. This supports the hypothesis that in these cells alternative termination of transcription may occur, just like in colorectal cancer cells investigated by Graham et al. [5]. Similarly, the 5′ UTRs of our transcripts may also be longer, considering the positive PCR results with the use of the CRNDEv2F primer (see Figure 3). This fact implies that *CRNDE* transcription in normal endometrium, ovarian cancer and HeLa cells starts at different initiation sites. Remarkably, we did not obtain expected PCR products for normal endometrium and ovarian cancer when using the CRNDEv1F primer, specific to exon 1a. The lack of amplification may suggest that transcripts containing this particular exon are not expressed in these tissues. On the other hand, the CRNDEv1F primer binds upstream of the main start sites identified by Graham et al. [5], so the existence of transcripts starting nearer the 3′ end of exon 1a cannot be excluded.

In summary, *CRNDE* seems to play a prominent role in carcinogenesis. While this study is the first demonstration of the clinical importance of *CRNDE* in cancer patients, other researchers also reported its oncogenic role in gliomas [6, 7, 16] and other solid tumors and leukemias [13]. Furthermore, *CRNDE* transcripts were implied to be of potential use as sensitive and specific molecular markers of early carcinogenesis in colon cancer [5]. With all this considered, this gene is likely to become in the future an important marker of cancer development or even a target for therapy.

## MATERIALS AND METHODS

### Patients and tumors

The first step of our study was the cDNA microarray-based evaluation of gene expression carried out in groups of 32 and 37 frozen tumor samples from ovarian cancer patients who underwent the cisplatin/cyclophosphamide (PC) or taxane/cisplatin (TP) treatment (see Table 2 for a detailed clinicopathological characteristics). Noteworthy, the microarray subgroup was selected from a larger tumor collection in such a way to minimize the influence of clinical factors on patients’ outcome. In other words, this group comprised the patients who had a good prognosis despite a large residual tumor (being a strong, negative prognostic factor), and *vice versa*. Next, we used Real-Time qPCR to verify these results in the same group of tumors. We also evaluated the clinical importance of *CRNDE* expression on 66 previously untested ovarian tumors from patients treated with TP, which gives 135 frozen tumor samples in total. All of them were collected prior to the chemical treatment in the Institute of Oncology, Warsaw, Poland in years 1995–2010. Medical records of all patients were critically reviewed by at least two clinicians. The material was carefully selected to meet the following criteria: no chemotherapy before staging laparotomy, adequate staging procedure, International Federation of Gynecologists and Obstetricians (FIGO) stage IIB to IV disease [24], tumor tissue from the first laparotomy available, moderate or poor tumor differentiation, availability of clinical data including residual tumor size and follow-up.

**Table 2:** Patients' characteristics

	Microarray group			Validation group
	PC regimen (*N* = 32)	TP regimen (*N* = 37)	PC+TP regimens (*N* = 69)	TP regimen (*N* = 66)
**Age of patients (years)**				
Range (median)	34–68 (54)	32–74 (53)	32–74 (53)	20–79 (53)
**Histological type**				
Serous	31 (96.9%)	30 (81.1%)	61 (88.4%)	49 (74.2%)
Endometrioid	-	2 (5.4%)	2 (2.9%)	2 (3.0%)
Clear-cell	-	-	-	3 (4.6%)
Undifferentiated	-	4 (10.8%)	4 (5.8%)	4 (6.1%)
Other types	1 (3.1%)	1 (2.7%)	2 (2.9%)	8 (12.1%)
**Histological grade**				
G1, G2	4 (12.5%)	5 (13.5%)	9 (13.0%)	9 (13.6%)
G3	18 (56.3%)	23 (62.2%)	41 (59.4%)	37 (56.1)
G4	10 (31.3%)	9 (24.3%)	19 (27.5%)	20 (30.3%)
**Clinical stage (FIGO)**				
IIB, IIC	-	2 (5.4%)	2 (2.9%)	1 (1.5%)
IIIA, IIIB	6 (18.8%)	5 (13.5%)	11 (15.9%)	4 (6.1%)
IIIC	22 (68.8%)	25 (67.6%)	47 (68.1%)	59 (89.4%)
IV	4 (12.5%)	5 (13.5%)	9 (13.0%)	2 (3.0%)
**Residual tumor size**				
0 cm	8 (25.0%)	7 (18.9%)	15 (21.7%)	18 (27.7%)*
≤2 cm	9 (28.1%)	23 (62.2%)	32 (46.4%)	35 (53.9%)
>2 cm	15 (46.9%)	7 (18.9%)	22 (31.9%)	12 (18.5%)
**Overall survival (days)**				
Range (median)	104–3750 (1165.5)	357–5630 (1069)	104–5630 (1111)	296–5569 (1161.5)
**Disease free survival (days)**				
Range (median)	97–2521 (369.5)	116–2452 (448)	97–2521 (416)	96–2884 (490)
**Outcome**				
NED	1 (3.1%)	3 (8.1%)	4 (5.8%)	10 (15.2%)
AWD	-	-	-	3 (4.6%)
DOD	31 (96.9%)	34 (91.9%)	65 (94.2%)	53 (80.3%)
**TP53 accumulation**				
TP53(−)	11 (34.4%)	14 (37.8%)	25 (36.2%)	24 (36.4%)
TP53(+)	21 (65.6%)	23 (62.2%)	44 (63.8%)	42 (63.6%)

NED – no evidence of disease; AWD – alive with disease; DOD – died of disease.

* The information on a residual tumor size for one patient from the validation group is missing.

All tumors were uniformly reviewed histopathologically, classified according to the criteria of the World Health Organization [25] and graded in a four-grade scale, in compliance with the standards given by Barber et al. [26]. Additionally, a complete evaluation of TP53 status has been performed using the PAb1801 mouse monoclonal antibody (1:500, Sigma-Genosys, Cambridge, UK), as described previously [10]. Accumulation of TP53 in immunohistochemical staining results predominantly from missense mutations (they account for about 67% of all TP53 alterations). In case of other mutations in the gene and when no alterations occur, accumulation of the TP53 protein is not observed [27]. From among 135 specimens that we examined, the TP53 accumulation occurred in 86 tumors (63.7%), while 49 tumors (36.3%) were TP53-negative.

As to the evaluation of clinical endpoints, complete remission (CR) was defined as disappearance of all clinical and biochemical symptoms of ovarian cancer evaluated after completion of the first-line chemotherapy and confirmed four weeks later [28]. Disease-free survival (DFS) time was assessed only for patients who achieved complete remission. For the PC-treated group, the follow-up time ranged from 104 to 3750 days (the median equaled 1165.5 days); the respective values for the TP-treated group were 296 and 5630 days (with the median of 1105 days). All surviving patients had at least a 6-month follow-up.

### RNA extraction followed by the assessment of its quantity and quality

RNA was isolated from frozen tumor sections with stromal cell contamination (scc) lower than 15% utilizing the RNeasy Plus Mini Kit (Qiagen, Hilden, Germany). RNA quantity was measured with NanoDrop specrophotometer (Thermo-Fisher Scientific, Waltham, MA, USA), and its quality was assessed on Agilent Bioanalyzer (Agilent Technologies, Santa Clara, CA, USA). RNA integrity numbers (RINs) of the samples ranged from 6.5 to 9.4.

### A microarray analysis of gene expression

Hybridizations were carried out as described by Lisowska et al., [29]. Briefly, double-stranded cDNA was synthesized with Reverse Transcriptase II (Life Technologies, Carlsbad, CA, USA) using 8 μg of total RNA as a template. Obtained cDNA (16 μg) was then used for the synthesis of biotinylated cRNA with the BioArray High Yield RNA Transcript Labeling Kit (Enzo Life Sciences, Farmingdale, NY, USA). Both cDNA and cRNA were purified with GeneChip Sample Cleanup Module (Affymetrix, Santa Clara, CA, USA). Fragmented cRNA was hybridized first to the Affymetrix control Test3 Array, and then, after evaluating the samples’ quality, to the Human Genome U133 Plus 2.0 Array (Affymetrix) for 16 h at 45°C. The microarrays were stained, washed, and subsequently scanned with GeneChip Scanner 3000 (Affymetrix). Data were acquired using the GCOS 1.2 software (Affymetrix).

### PCR experiments

All polymerase chain reactions (PCRs) were run on cDNA using the AmpliTaq Gold DNA Polymerase (Life Technologies). They consisted of the following steps: 95°C for 10 min. (hot start of the polymerase), 95°C for 30 sec. (DNA denaturation), 55°C for 30 sec. (primers annealing), 72°C for 1 min. (the PCR product elongation), 72°C for 7 min. (final elongation of the product), 4°C hold. DNA denaturation, primers annealing and product elongation steps were repeated consecutively 35 times. The ND (no-DNA) sample was used as a negative reference in every PCR reaction. Sequences of all PCR primers are shown in Table S3.

### Development of two TaqMan assays for two different splice variants of *CRNDE*

Two different sets of primers (LOCrtF, LOCrt35R) and (LOCrtF, LOCrt4wR) as well as one TaqMan probe were designed and tested, according to PE Biosystems’ recommendations. These assays were able to specifically detect either the short or the long splice variant of *CRNDE* (see Figure S5), though the same TaqMan probe (27 bp) was utilized in both cases. This was achieved by using either the LOCrt35R or LOCrt4wR reverse primer. The former primer spanned a junction between exon 4 and the shorter splice variant of exon 5, which made it specific to the FJ466686 transcript. On the contrary, the LOCrt4wR primer was fully homologous to exon 5 in a region present in FJ466685 and absent in the FJ466686 transcript (see Figure 4). The genomic sequence of *CRNDE* was undetectable by both primer sets due to the presence of large introns (> 4 kb) between the amplified exons.

Both personally designed TaqMan assays specific to the *CRNDE* gene were thoroughly tested before use, as suggested by PE Biosystems [22], to ensure the best specificity and performance of Real-Time qPCR. Results of the specificity tests are presented in Figure S5, whereas the performance of each assay equaled about 100% (assessed based on a slope of the standard curve). In addition, the correctness of both Real-Time qPCR products was confirmed by DNA sequencing with the use of BigDye Terminator v3.1 Cycle Sequencing Kit (Life Technologies).

### Real-time qPCR-based studies of gene expression

Gene expression data obtained from cDNA microarrays were further verified by Real-Time qPCR. This study was performed not only in the microarray group, but also on another set of 66 previously untested ovarian tumors (so-called the validation group) in order to confirm a clinical significance of the results. All Real-Time qPCR experiments described here were run in triplicates on the 7500 Fast Real-Time PCR System (Life Technologies), using *HGPRT* as a reference gene. Gene expression was evaluated with TaqMan assays, *CRNDE*-specific (6-FAM-labeled, personally designed) and *HGPRT*-specific (VIC-labeled, Life Technologies, assay id: 4326321E). All qPCR experiments were carried out as standard singleplex reactions in the volume of 10 μl using TaqMan Universal PCR Master Mix with uracil N-glycosylase (Life Technologies) and about 20 ng of total RNA, earlier reverse transcribed to cDNA with the High-Capacity cDNA Reverse Transcription Kit (Life Technologies). The expression of *CRNDE* was normalized to *HGPRT* and relatively quantified using the delta-delta-CT algorithm [30].

The reference gene used in this study, *HGPRT*, was nominated from among 11 genes included on TaqMan Human Endogenous Control Plates (Life Technologies), because it was characterized by the most stable expression in both the PC- and TP-treated groups. Expression of the reference genes was assessed for 8 randomly selected tumors from each group. Then, the stability was calculated with the qBase^PLUS^ software (Biogazelle NV, Zwijnaarde, Belgium), utilizing an improved version of the geNorm algorithm [31, 32].

### Statistical analyses of gene expression data

Microarray data were subjected to a statistical analysis utilizing the GC-RMA algorithm [33]. Further noise reduction was achieved by the elimination of the least significant principal components. Quality control parameters were based on MAS5.0-extracted information, using thresholds suggested by Affymetrix [34]. Internal consistency of the data sets was also tested using the principal component analysis (PCA). A selection of differentially expressed genes was carried out using the resampling-based test with t statistics. Probe sets with *p*-values ≤ 0.001 and fold change (FC) values ≥1.5 were considered significantly changed.

A prognostic value of the data obtained in microarray and qPCR experiments was further verified using the stepwise multivariate Cox proportional hazards model. In this analysis, the following 6 variables were taken into account: gene expression level, patient's age (categorized by median split); residual tumor size; clinical stage (FIGO); histological grade (the last three parameters were categorized as shown in Table 2) and histological type (categorization: serous vs non-serous types). The survival analyses were performed not only in the entire group of tumors, but also in subgroups with and without accumulation of TP53. Since the PC-treated group was too small for a multivariate analysis, the univariate Cox proportional hazards model was applied to it. In addition, we conducted the statistical inference in the joined PC+TP-treated groups to find out whether the outcome depends on the therapeutic regimen used, and if this effect is additive or subtractive.

The association of *CRNDE* expression with either clinicopathological parameters or the TP53 status in the tumors was assessed using the Mann-Whitney U test or the Kruskal-Wallis test, respectively, depending whether the nominal variable had two or more categories.

Herein, the expression of *CRNDE* was always treated as a continuous variable to avoid arbitrary categorization of data, which could potentially lead to falsification of statistical results. It is worth noting that, in case of continuous variables, HRs cannot be treated as the ratio of the hazard rates, corresponding to the conditions described by two sets of explanatory variables (as for categorical variables), because these sets do not exist. For continuous variables, the same interpretation applies to a unit difference [35]. In our study, a tumor exhibiting the highest expression of each *CRNDE* transcript was used as a calibrator. Thus, all the expression values ranged from 0 to 1. This approach allowed for approximate estimation of the risk of death and recurrence based on the hazard ratio (HR) values in a similar way as for categorical variables. However, it has to be stressed that the real HR will always be lower from what is shown in Tables 1 and S2, because only one tumor (calibrator) has the *CRNDE* expression equaling 1, and none – equaling 0.

## SUPPLEMENTARY DATA, FIGURES AND TABLES


