# Supplementary material for: The putative oncogene, CRNDE, is a negative prognostic factor in ovarian cancer patients
Source: Oncotarget. 2015 Nov 4;6(41):43897–910. doi: 10.18632/oncotarget.6016 (PMC4791275; doi:10.18632/oncotarget.6016)
Supplement: Supplementary file 1 [file oncotarget-06-43897-s001.pdf]

SUPPLEMENTARY DATA

Rapid amplification of cDNA ends (RACE)

The 3′ RACE reaction was performed on one RNA sample isolated from normal human endometrium. RNA was reverse transcribed with the use of Superscript III First − Strand Synthesis System (Life Technologies), as recommended by the manufacturer, but with one prominent modification: the oligo(dT)20 primer was replaced with the oligo(dT) anchor primer (5′/3′ RACE Kit; Roche,). In this step, 1.4 μg of RNA and 37.5 pmol of the mentioned anchor primer were used. A subsequent PCR reaction was run in 25 μl using the following primers: LOC388 − F and PCR anchor primer (5′/3′ RACE Kit; Roche) (see Figure S3), according to the standard PCR protocol with the following modifications: primers annealing temperature was raised to 65°C and elongation time was prolonged to 2 minutes. The PCR products were separated by cloning them into *Escherichia coli* TOP10 strain in the pGEM − T Easy vector (Promega, Fitchburg, WI, USA). Plasmids with a correct *EcoRI* restriction pattern were sequenced and obtained sequences were aligned to the reference sequence of chromosome 16 (see Figure S1).

The 5' RACE reaction was run with the use of FirstChoice RLM-RACE kit (Ambion, Carlsbad, CA, USA). The CIP/TAP treatment, ligation of the 5' RACE RNA adapter (Ambion) and reverse transcription were performed according to the manufacturer's recommendations. Next, 3 successive nested PCR reactions were run, using three different sets of primers (see Figure S4). In all reactions, primers annealing temperature was set to 60 °C and elongation time to 3 minutes to allow amplification of products up to 3 kb. The sequence of the final product of nested PCR was aligned to the reference sequence of chromosome 16 (see Figure S2).

RACE studies revealed the existence of two novel splice variants of the *CRNDE* gene

In 3′ RACE studies, we identified three different products, each consisting of approximately 500 − 600 bp. The products were cloned into E. coli cells. Five of eight clones had proper DNA inserts of either the shorter or the longer CRNDE splice variant (see Figure S1). The remaining three clones contained a fragment of the *Homo sapiens* ribosomal protein S2 (RPS2) gene, encoding a component of the ribosomal 40S subunit. Apparently, 11 bp-homology of the LOC388-F primer to the aforementioned gene was sufficient to cause mispriming. Remarkably, one of five proper clones contained the CRNDE transcript with the 3' UTR region longer by 5 nucleotides than the others, which may suggest the existence of mechanisms of alternative transcription termination.

5' ends of CRNDE transcripts were investigated via RLM-RACE followed by 3 nested PCR reactions (see Figure S2), which resulted in a single product consisting of 216 bp. The products of 3' RACE and 5' RACE experiments had a common region of 82 bp, which proved the completeness of both CRNDE transcripts identified herein. We submitted their sequences to GenBank in November 2008, where they were assigned the accession numbers FJ466685 and FJ466686.

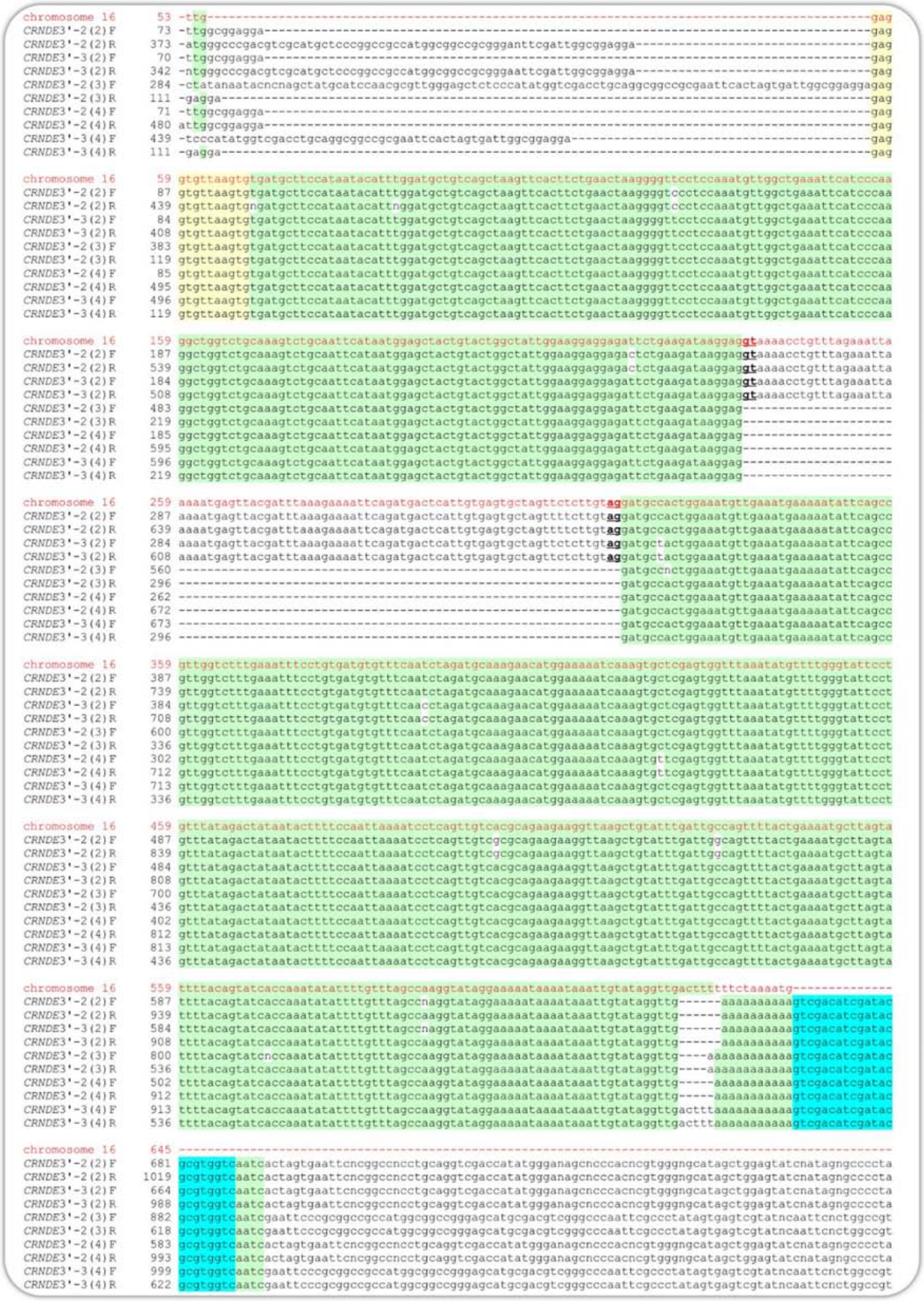

Supplementary Figure S1: An alignment of *CRNDE* cDNA sequences from five different *E. coli* clones identified after 3′ RACE. Each clone was sequenced twice from either the forward (F) or the reverse (R) primer. A reference sequence of chromosome 16 was marked red. A partial sequence of the LOC388-F primer (13 bp) was highlighted in yellow and the PCR anchor primer (Roche) – in blue. GT-AG sequences that delimit human introns were written in bold letters. The CRNDE3′-3(4) clone had a bit longer 3′ UTR than the others.

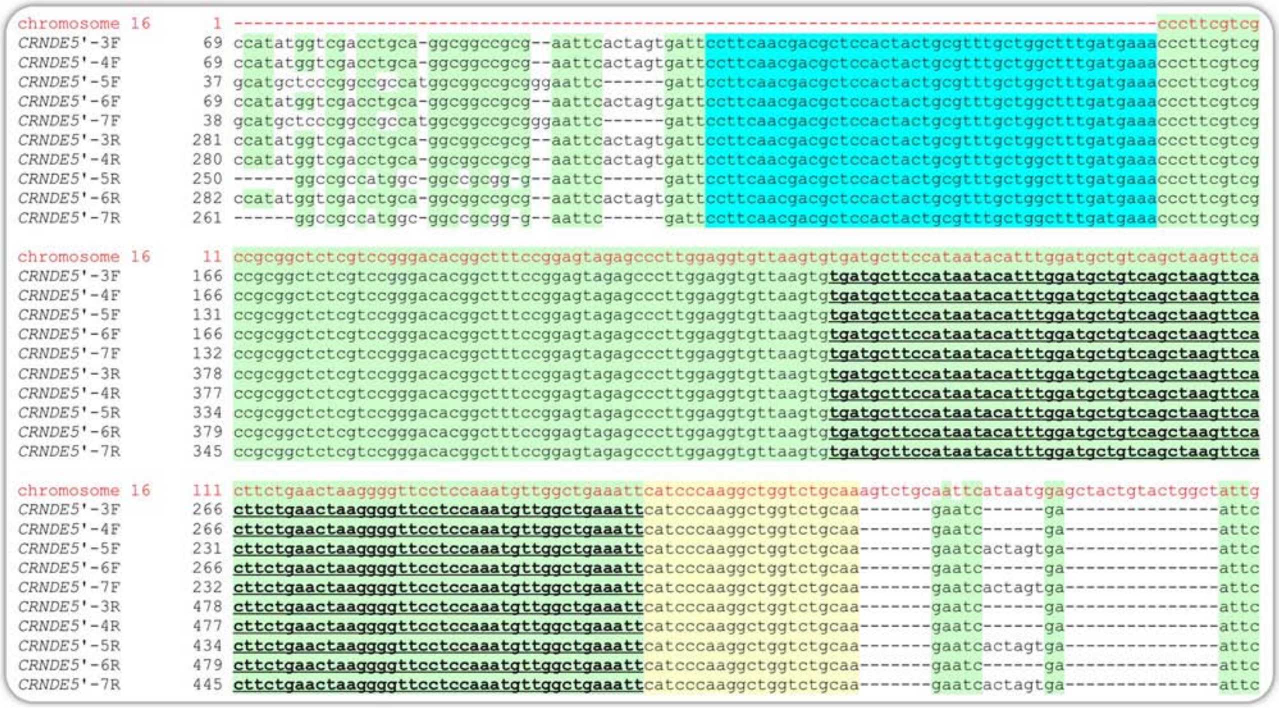

Supplementary Figure S2: An alignment of *CRNDE* cDNA sequences from five different *E. coli* clones identified after 5′ RACE. Each clone was sequenced twice from either the forward (F) or the reverse (R) primer. A reference sequence of chromosome 16 was marked red. The overlapping sequence (82 bp long, identified in both RACE experiments) was written in bold, underlined letters. The sequence attached to the 5′ end of *CRNDE* RNAs was highlighted in blue and the LOC388-R primer – in yellow.

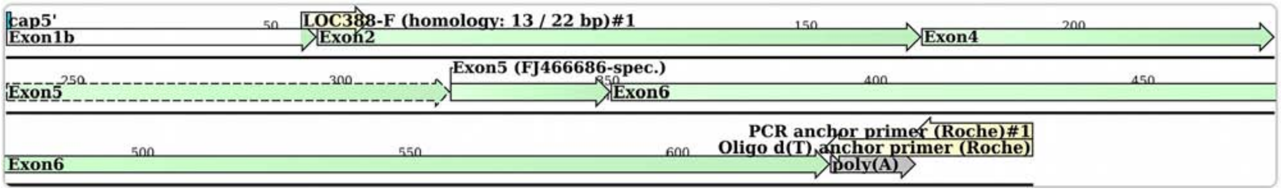

Supplementary Figure S3: A diagram of the 3′ RACE experiment. The primers used in the subsequent nested PCR reaction were numbered (#1). Their sequences are specified in Table S3.The alternatively spliced exon 5 was indicated with a dashed line, while the unidentified region of the transcripts remained uncolored.

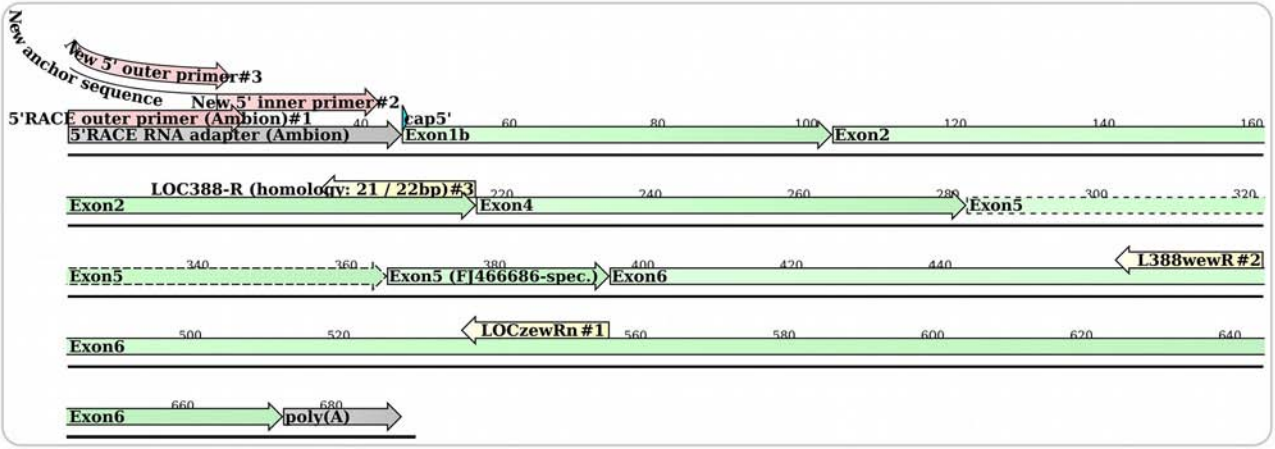

Supplementary Figure S4: A diagram of the 5′ RACE experiment. The primers used in three successive nested PCR reactions were marked with the same number (e.g. #1). Their sequences are specified in Table S3. The alternatively spliced exon 5 was indicated with a dashed line.

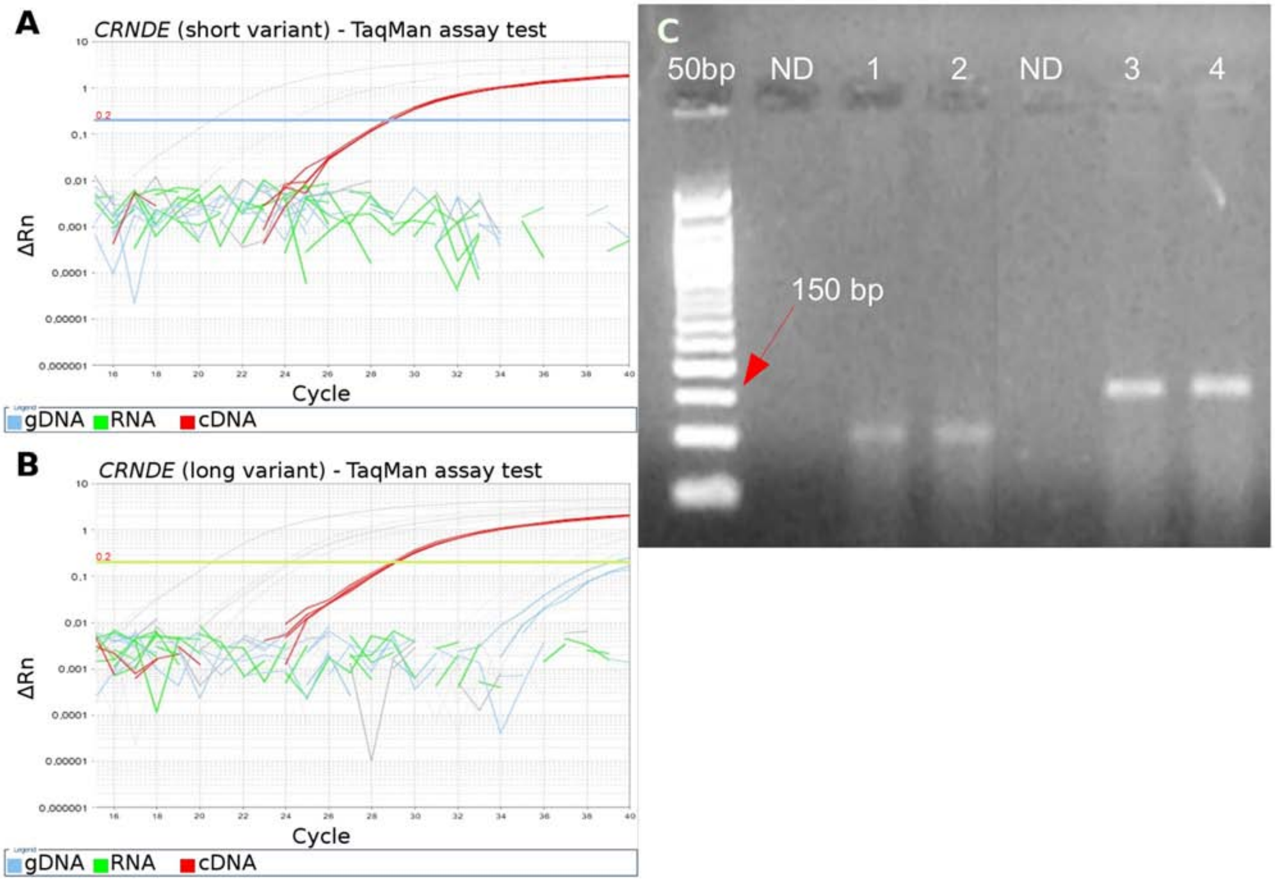

Supplementary Figure S5: A verification of specificity of two Real-Time qPCR TaqMan assays. Both assays for either the short **A.** or the long **B.** *CRNDE* splice variant amplified specifically only the cDNA templates. There was no signal from genomic DNA or RNA templates. Next, the Real-Time qPCR products were verified by agarose gel electrophoresis **C.** showing the correct length of both amplicons, i.e., 104 bp (lanes 1–2) and 160 bp (lanes 3–4) for the short and the long splice variant, respectively. 50 bp – 50 bp DNA ladder; ND – no-DNA control.

Supplementary Table S1: Selected results of our microarray studies preceding the Real-Time qPCR-based evaluation of *CRNDE* expression

GEO:GSE63885 microarrays, the TP-treated group, *N* = 37, short vs long overall survival (OS)						
**Probe set**	**GenBank**	**Symbol**	**Short OS**	**Long OS**	**FC**	***P*-value**
**238022_at**	**AA954994**	**CRNDE**	**466.412**	**93.301**	**4.999**	**0.00047013**
231729_s_at	NM_004058	CAPS	899.251	247.966	3.627	0.00087401
226622_at	AW084511	MUC20	867.363	349.505	2.482	0.00041909
223285_s_at	AW044319	ST6GALNAC4	237.287	122.814	1.932	0.00043374
209260_at	BC000329	SFN	391.746	203.879	1.921	0.00054964
228307_at	AL137580	EMILIN3	346.127	207.395	1.669	0.00032412
205536_at	NM_003371	VAV2	306.7	199.528	1.537	0.0003819
227802_at	AI075999	RUFY3	213.432	328.85	0.649	0.00069531
219166_at	NM_018139	DNAAF2	344.805	534.437	0.645	0.00079554
210320_s_at	AF077033	DDX52	276.38	441.325	0.626	0.00014829
224765_at	AA001203	MSL1	1438.326	2342.193	0.614	0.00024317
204808_s_at	NM_014254	TMEM5	1090.427	1832.357	0.595	0.00049784
228066_at	AI870951	C17orf96	198.631	339.076	0.586	2.909e-05
219311_at	NM_024899	CEP76	251.273	428.992	0.586	0.00053877
204934_s_at	NM_002151	HPN	240.597	490.878	0.49	0.00074734
209900_s_at	AL162079	SLC16A1	194.65	429.725	0.453	0.00093968
219683_at	NM_017412	FZD3	228.609	577.952	0.396	0.00019386
**GEO:GSE63885 microarrays, the TP-treated group with TP53 accumulation, *N* = 23, short vs long OS**						
**Probe set**	**GenBank**	**Symbol**	**Short OS**	**Long OS**	**FC**	***P*-value**
**238022_at**	**AA954994**	**CRNDE**	**392.656**	**68.636**	**5.721**	**0.00090405**
**238021_s_at**	**AA954994**	**CRNDE**	**1065.614**	**189.93**	**5.611**	**0.00069111**
223894_s_at	BC001134	FTS	640.32	341.174	1.877	0.00085233
218373_at	NM_022476	FTS	1065.72	596.247	1.787	0.00089059
222994_at	AF197952	PRDX5	3784.257	2413.708	1.568	0.00036439
202120_x_at	NM_004069	AP2S1	758.107	1139.827	0.665	0.000124
211047_x_at	BC006337	AP2S1	1795.773	2850.67	0.63	2.353e-05
206303_s_at	AF191653	NUDT4	344.145	548.019	0.628	0.00077233
203832_at	NM_003095	SNRPF	1319.418	2185.298	0.604	0.00071822
220160_s_at	NM_007059	KPTN	116.313	206.693	0.563	0.00020395
210320_s_at	AF077033	DDX52	285.703	515.121	0.555	0.00085172
202875_s_at	BE397715	PBX2	359.196	679.579	0.529	9.511e-05
202094_at	AA648913	BIRC5	212.2	418.344	0.507	0.0003439
203588_s_at	BG034328	TFDP2	533.847	1134.614	0.471	0.0007539
219683_at	NM_017412	FZD3	193.443	427.626	0.452	0.00091274
227230_s_at	BE855799	KIAA1211	242.532	602.054	0.403	0.00094198

Only the results with *p*-values < 0.001 and Fold Change (FC) higher than 1.5 are shown. In the survival analysis, CRNDE was the gene with the highest FC in both the entire TP-treated group of ovarian cancer patients, and in the subgroup with TP53 accumulation. Data are sorted according to the decreasing FC value. The results for probe sets 238021_s_at and 238022_at, which are specific to the CRNDE gene, are written in bold letters.

Supplementary Table S2: Evaluation of a prognostic value of the *CRNDE* gene expression in the microarray and validation groups of ovarian cancer patients treated with TP. The multivariate Cox proportional hazards model was utilized in this analysis

Microarray group – TP regimen						
			The TP53 (+) subgroup		The TP53 (−) subgroup	
	OS (34/37)*	DFS (20/22)*	OS (20/23)*	DFS (15/17)*	OS (14/14)*	DFS (5/5)*
Variable name	HR [95% CI] p	HR [95% CI] p	HR [95% CI] p	HR [95% CI] p	HR [95% CI] p	HR [95% CI] p
CRNDE (short variant) high vs low expr.	8.044 [1.709 – 37.86] 0.008	18.03 [3.020 – 107.7] 0.002	27.44 [3.405 – 221.1] 0.002	NS	NS	NS
Rt ≤ 2cm vs 0cm	2.625 [1.069 – 6.443] 0.035	–	–			
Grade 3 vs (1+2)	3.545 [1.054 – 11.93] 0.041	–	–			
Grade 4 vs (1+2)	5.106 [1.280 – 20.38] 0.021	–	–			
CRNDE (long variant) high vs low expr.	13.34 [2.005 – 88.74] 0.007	17.47 [2.243 – 136.2] 0.006	68.11 [4.110 – 1129] 0.003	NS	NS	NS
Type (serous vs non – serous)	–	6.348 [0.768 – 52.49] 0.086	–			
Rt ≤ 2cm vs 0cm	2.616 [1.075 – 6.370] 0.034	–	–			
Grade 3 vs (1+2)	3.405 [1.007 – 11.51] 0.049	–	–			
Grade 4 vs (1+2)	6.017 [1.505 – 24.06] 0.011	10.71 [1.626 – 70.56] 0.014	–			
**Validation group – TP regimen**						
CRNDE (short variant) high vs low expr.	NS	39.95 [0.559 – 2854] 0.090	NS	NS	NS	> 9999 [107.4 – > 9999] 0.005
Age ≥ 53 vs <53 years		2.059 [1.000 – 4.239] 0.050				11.40 [1.976 – 65.77] 0.006
**Validation group – TP regimen**						
Rt >2cm vs 0cm		2.653 [0.846 – 8.322] 0.094				79.24 [6.286 – 999.0] 0.001

(*Continued*)

**Rt ≤2cm vs 0cm**		2.273 [1.063 – 4.861] 0.034				3.852 [0.856 – 17.33] 0.079
**Grade 3 vs (1+2)**		2.178 [1.076 – 4.410] 0.030				–
**Grade 4 vs (1+2)**		–				281.6 [0.469 – > 9999] 0.084
***CRNDE* (long variant) high vs low expr.**	NS	NS	NS	NS	88.06 [0.977 – 7938] 0.051	5301 [4.397 – > 9999] 0.018
**Age ≥ 53 vs <53 years**					3.987 [1.234 – 12.88] 0.021	7.933 [1.850 – 34.01] 0.005
**Type (serous vs non – serous)**					3.773 [0.916 – 15.54] 0.066	–
**Rt >2cm vs 0cm**					9.432 [1.859 – 47.85] 0.007	7.151 [1.080 – 47.35] 0.041
**Grade 3 vs (1+2)**					–	4.237 [1.176 – 15.27] 0.027
**Grade 4 vs (1+2)**					16.91 [2.763 – 103.5] 0.002	79.73 [3.322 – 1913] 0.007

[*] Values before and after a slash (/) stand for the number of completed observations vs all observations, respectively. Only the expression results with p –values < 0.1 are shown and those with p-values < 0.05 are underlined. HR and CI stand for the hazard ratio and 95% confidence interval, respectively. NS – a non-significant result.

Supplementary Table S3: PCR primers and other synthetic DNA and RNA molecules used in the present study

Primer name	Sequence [5′ → 3′]
**5′ RACE RNA adapter** (FirstChoice RLM − RACE Kit, Ambion)	GCUGAUGGCGAUGAAUGAACACUGCGUUUGCUGGCUUUGAUGAAA
**5′ RACE outer primer** (FirstChoice RLM − RACE Kit, Ambion)	GCTGATGGCGATGAATGAACACTG
**New 5′ outer primer**	CCTTCAACGACGCTCCACTAC
**New 5′ inner primer** (with a new anchor sequence)	CCTTCAACGACGCTCCACTACTGCGTTTGCTGGCTTTGATG
**PCR anchor primer** (5′/3′ RACE Kit (Roche)	GACCACGCGTATCGATGTCGAC
**Oligo d(T) anchor primer** (5′/3′ RACE Kit, Roche)	GACCACGCGTATCGATGTCGACTTTTTTTTTTTTTTTTV[1]
***CRNDE*-specific TaqMan probe** (universal)	CCTTCCAATAGCCAGTACAGTAGCTCC
**LOCrtF** – Real-Time PCR forward primer (universal)	AATTCATCCCAAGGCTGGTC
**LOCrt35R** – Real-Time PCR reverse primer (specific to the CRNDEP-coding transcript, FJ466686)	TTCCAGTGGCATCCTCCTTA
**LOCrt4wR** – Real-Time PCR reverse primer (specific to non-CRNDEP-coding transcripts, e.g., FJ466685)	GCACTCACAATGAGTCATCTG
**CRNDEv1F** PCR primer	TGGCGCTAACGGTCGGTAAC
**CRNDEv2F** PCR primer	TCTGTCCACGCCTGTTCCCTTC
**LOC388-F** PCR primer	GGCGGAGGAGAGGTGTTAAGTG
**LOC388-R** PCR primer	CTTGCAGACCAGCCTTGGGATG
**L388zewR** PCR primer	TCAGGTATAAACATTCATTC
**LOCzewRn** PCR primer	CTTCTGCGTGACAACTGAGG
**L388wewF** forward sequencing primer	CTGTACTGGCTATTGGAAGG
**L388wewR** reverse sequencing primer	TAAACCACTCGAGCACTTTG

[1] V stands for A or C or G, according to IUPAC nucleotide ambiguity codes.
